# Selenocystine-Derived Label-Free Fluorescent Schiff Base Nanocomplex for siRNA Delivery Synergistically Kills Cancer Cells

**DOI:** 10.3390/molecules27041302

**Published:** 2022-02-15

**Authors:** Yang Liu, Haoying Yang, Qian Liu, Mingming Pan, Danli Wang, Shiyuan Pan, Weiran Zhang, Jinfeng Wei, Xiaowei Zhao, Junfeng Ji

**Affiliations:** 1 Center of Stem Cell and Regenerative Medicine, School of Medicine, Zhejiang University, Hangzhou 310058, China; liuyang_bms@zju.edu.cn; 2 Henan Key Laboratory of Brain Targeted Bio-Nanomedicine, School of Life Sciences & School of Pharmacy, Henan University, Kaifeng 475004, China; yhaoy98@163.com (H.Y.); liuqian.research@outlook.com (Q.L.); mingmpan@outlook.com (M.P.); pan353491@163.com (S.P.); weiran3986@163.com (W.Z.); 3 Zhoushan Hospital of Zhejiang Province, Zhoushan 316004, China; 15395823211@163.com

**Keywords:** selenocystine, fluorescent Schiff base, Schiff base probe, siRNA nanoparticle, tumor senescence

## Abstract

Chemo and siRNA synergic treatments for tumors is a promising new therapeutic trend. Selenocystine, a selenium analog of cysteine, has been considered a potential antitumor agent due to its redox perturbing role. In this study, we developed a nanocarrier for siRNA based on a selenocystine analog engineered polyetherimide and achieved traceable siRNA delivery and the synergic killing of tumor cells. Notably, we applied the label-free Schiff base fluorescence mechanism, which enabled us to trace the siRNA delivery and to monitor the selenocystine analogs’ local performance. A novel selenocystine-derived fluorescent Schiff base linker was used to crosslink the polyetherimide, thereby generating a traceable siRNA delivery vehicle with green fluorescence. Moreover, we found that this compound induced tumor cells to undergo senescence. Together with the delivery of a siRNA targeting the anti-apoptotic *BCL-xl/w* genes in senescent cells, it achieved a synergistic inhibition function by inducing both senescence and apoptosis of tumor cells. Therefore, this study provides insights into the development of label-free probes, prodrugs, and materials towards the synergic strategies for cancer therapy.

## 1. Introduction

Selenocystine (SeC) is a diselenide compound and selenium analog of cysteine but is more reactive than cystine due to the chemical property of selenium. Previous studies have implied selenocystine’s antineoplastic potentials since it can trigger a variety of cell cycle arrests and apoptotic responses [1,2,3,4,5,6,7,8]. Mechanistically, selenocystine can be reduced by glutathione (GSH), thioredoxin reductase, or excess cysteine to highly reactive selenolate, which generates oxidative stress and perturbs redox homeostasis in the cell [9]. SeC is also a naturally occurring selenoamino acid, which has been shown to be well tolerated in rodent models and normal cells and has been applied in many preclinical studies [10,11]. Tumor cells generally exhibit an elevated GSH level, which adjusts the hyper redox perturbation during tumorigenesis [12]. Therefore, a broad-spectrum antitumor application of SeC may be achieved. Nanodelivery technology facilitates the synergetic therapy by both chemo agents and siRNA, which has been a promising new trend towards achieving better therapeutic effects and less drug resistance for multiple diseases [13,14,15,16]. siRNA-based therapeutics have rapidly crowed into clinical trials, and some of them have already been approved for the market due to the relatively higher specificity and ease of synthesis [17]. However, challenges for siRNA therapeutics lie in the effective delivery and enhancing the treatment effects [18]. Emerging studies have demonstrated that novel nanotech-based strategies can robustly enhance drug and siRNA delivery, which offers synergetic treatment potentials for multiple diseases [19,20,21,22,23,24]. Cationic polymeric vectors are the most studied siRNA delivery vectors [25]. Nevertheless, difficulties, such as high cytotoxicity, low transfection efficiency, and uncontrollable and untraceable gene transfer, still hamper its progression into clinical applications of gene therapy [26]. The high cytotoxicity of a cationic vector is generally attributed to the plentiful positive charge and non-biodegradable accumulation of the vector [21,27]. Low delivery efficiency and poor vector unpacking are the major limiting steps for high-performance gene delivery. Therefore, the attainment of gene delivery systems that are safe, highly efficient, smart, and multiple-functional is imperative.

In order to reduce high cytotoxicity and to improve the gene release efficiency, stimuli-responsive delivery systems have been proposed, where “smart” vectors can load the siRNA and then be degraded by external stimuli, such as pH, temperature, enzymes, and microenvironment [28,29,30,31]. It is necessary to develop biocompatible and biodegradable vectors, which can be triggered by the biological environment. The S-S bond in cystine and Se-Se bond in selenocystine were reported to respond to the GSH in tumors [30,32], which offers a controlled release option.

siRNA transfection to the target cells is an invisible multiple-step process. In order to monitor the delivery and release process, fluorochromes are commonly used to label the chemicals or siRNAs [33]. However, fluorochromes may change the chemical structure and properties of the vectors, thereby disturbing the real physical/chemical state compared with label-free probes. In addition, fluorochromes are also implicated with high cost, safety concerns, and disposal problems. Therefore, traceable delivery vectors with label-free fluorescence and the controlled release for siRNA are needed. In order to achieve this, the Schiff base (-N=CR-) fluorescence mechanism may be helpful. Schiff base is a very useful organic chemical structure that is involved in click chemistry, battery, catalysts, intermediates in polymer stabilizers, organic synthesis, self-recovery, biomedical compounds, bioprinting, and biosensors design [34,35]. Interestingly, studies have reported that the Schiff base derived from acetaldehyde reacts with cystine and possesses fluorescence [36,37]. This phenomenon implies that the similar structure in selenocystine that reacts with acetaldehyde may also exhibit fluorescence. Selenocystine is also capable of reacting with the NH_2_-rich structure of branched polyetherimide (bPEI) to reduce the positive charge, thus decreasing cytotoxicity. Moreover, the Se-Se bond may have a stronger response to the tumor microenvironment compared to S-S bond and plays an enhanced anti-tumor role due to the redox perturbing capacity.

**Figure 1:**
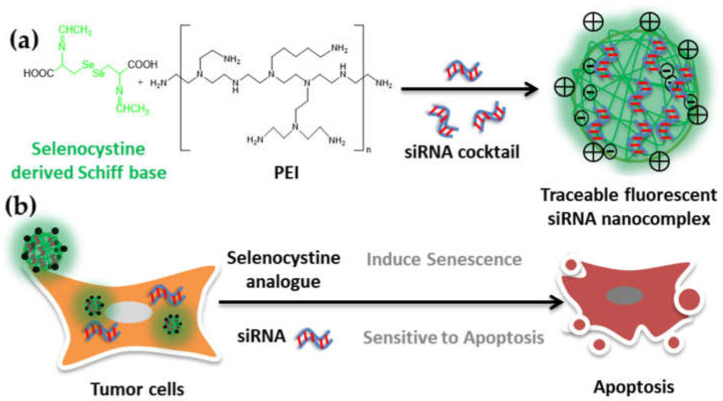
Schematic showing the development process of a traceable fluorescent delivery vehicle for siRNA and its application in synergic elimination of tumor cells. (**a**) In this study, selenocystine-derived Schiff base exhibiting a fluorescent activity was identified for the first time. Furthermore, as a linker, the selenocystine Schiff base analog was used to conjunct PEI for siRNA loading and tumor treatment. (**b**) siRNA cocktail targeting *BCL-w/xl* was loaded by the traceable fluorescent siRNA vehicle and was delivered to the HepG2 tumor cells. Selenocystine analog induced the HepG2 tumor cells to undergo senescence with an elevated ROS level. Moreover, the imbalanced hyperactive redox microenvironment of tumor cells triggers the siRNA release and sensitizes tumor cells to apoptosis. The simple strained selenocystine-derived Schiff base with label-free fluorescent activity may offer broad potential for probe and traceable prodrug development.

In this study, in order to develop a traceable delivery vehicle for siRNA, a selenocystine-derived Schiff base exhibiting a fluorescent activity was identified for the first time. Furthermore, as a linker, the selenocystine Schiff base analog was used to conjunct PEI for loading siRNA targeting anti-apoptotic *BCL-w/xl* genes. The selenocystine analog induced the senescence of HepG2 tumor cells, and apoptosis was sequentially triggered by siRNA inhibiting apoptosis-resistant pathways in the senescent tumor cells, which enables a synergistic elimination (Figure 1).

## 2. Results

### 2.1. Developing Schiff Base Fluorescence Vector for siRNA

**Figure 2:**
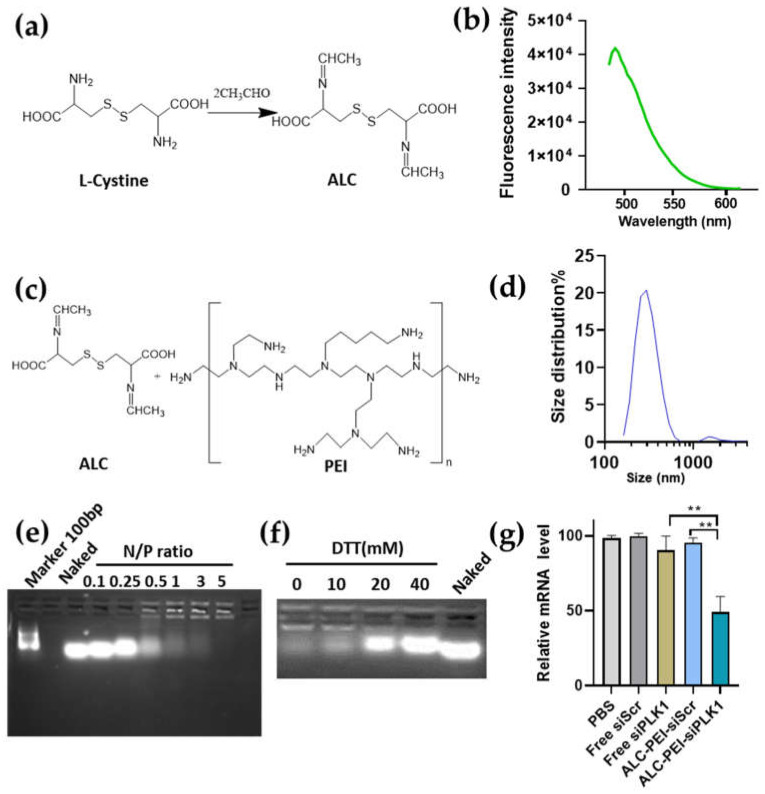
Schiff base compound derived from L-cystine reacts with acetaldehyde and mediates siRNA delivery. (**a**) Synthesis of the acetaldehyde-L-cysteine (ALC). (**b**) ALC fluorescence at excitation 460 nm. (**c**) Reaction for the structure unit in the ALC-PEI complex. (**d**) Size distribution of the ALC-PEI nanodelivery vector. (**e**) Gel retardance assay for siRNA loading. The different amine of bPEI (N) to the phosphate of siRNA (P) ratios (N:P) were used to indicate bPEI and siRNA ratios. Naked siRNA was noted as “naked.” (**f**) siRNA releasing under the stimuli of DTT (mM). (**g**) *PLK1* mRNA expression after different treatments. N:P = 5, 100 nM siRNA was used. Samples were collected 3 days post-treatment. Data were presented as mean ± SEM. ** *p* < 0.01.

Studies reported that the Schiff base containing a compound synthesized by aldehyde and L–cysteine (ALC) exhibits fluorescence at 462Ex (Excitation)/488Em (Emission) [36,37]. As a linker, ALC contains two –COOH groups, which can crosslink the –NH_2_ rich compound bPEI (branched Polyethyleneimine) to obtain a nanocomplex with nucleic acid loading capacity. To further apply this concept to siRNA loading, we first crosslinked the bPEI by ALC (ALC-PEI). PEI is a very common transfection reagent due to its high positive charge density, but it also brings toxicity to cells. The lower molecular weight branched PEI (such as bPEI 0.8k) exhibited a reduced cytotoxicity compared with linear PEI (lPEI) and was used to form the siRNA-PEI complex [25,26]. Firstly, we prepared the ALC and verified its fluorescence activity. In line with previous reports, our results showed that a fluorescence at 462Ex/488Em was detected (Figure 2a,b). We then crosslinked the fluorescent Schiff base linker, ALC, to bPEI, forming the ALC-PEI nanocomplex with a size distribution of around 200–500 nm (Figure 2c,d). siRNA was also successfully loaded by ALC-PEI at the ratio of N/P = 5 (the N/P ratio used was amine of bPEI (N) to the phosphate of siRNA (P) ratio) (Figure 2d). Moreover, the nanocomplex showed a release capacity responsive to treatment with the general reduction agent dithiothreitol (DTT) (Figure 2f). In order to evaluate the gene silencing ability of the developed ALC-PEI polymer as a siRNA carrier, siRNAs targeting the human polo-like kinase 1 (PLK1) gene as a test siRNA were delivered. The gene expression results confirmed that siPLK1 delivered by ALC-PEI significantly reduced the PLK1 mRNA expression in Kirsten human osteogenic sarcoma (KHOS) cells in comparison with the scramble control and free siPLK1 (Figure 2g). Since PLK1 is critical for KHOS cell proliferation, the successful intracellular delivery of siPLK1 can be monitored by the KHOS cells’ survival rate. Our data showed that the KHOS cell survival rate was maintained at over 99% when they were incubated with ALC-PEI alone and without siRNA (Figure S3, Supplementary Materials). By contrast, the commercial lPEI 22k without siPLK1 significantly decreased the cell survival rate of KHOS cells, indicating the cytotoxicity of IPEI. Our results showed that, while siPLK1 delivered by ALC-PEI significantly decreased the cell survival rate in a dose-dependent manner, the cell survival rate of KHOS cells was maintained at over 95% for bPEI 0.8k, even with the highest concentration of siPLK1 (150 nM), suggestive of bPEI’s weak siRNA loading capacity (Figure S3). When taken together, these results demonstrated that the ALC-PEI polymer could efficiently deliver siRNA into KHOS cells to silence gene expression. The effective performance of the developed ALC-PEI vector might be attributed to the enhanced siRNA loading when forming a nanocomplex and siRNA release triggered by the S-S bond in the intracellular microenvironment. The combination of these advantages suggests that a structure such as ALC-PEI may have more potential as a carrier for siRNA delivery.

### 2.2. Selenocystine-Derived Schiff Base Exhibits a Fluorescent Activity

**Figure 3:**
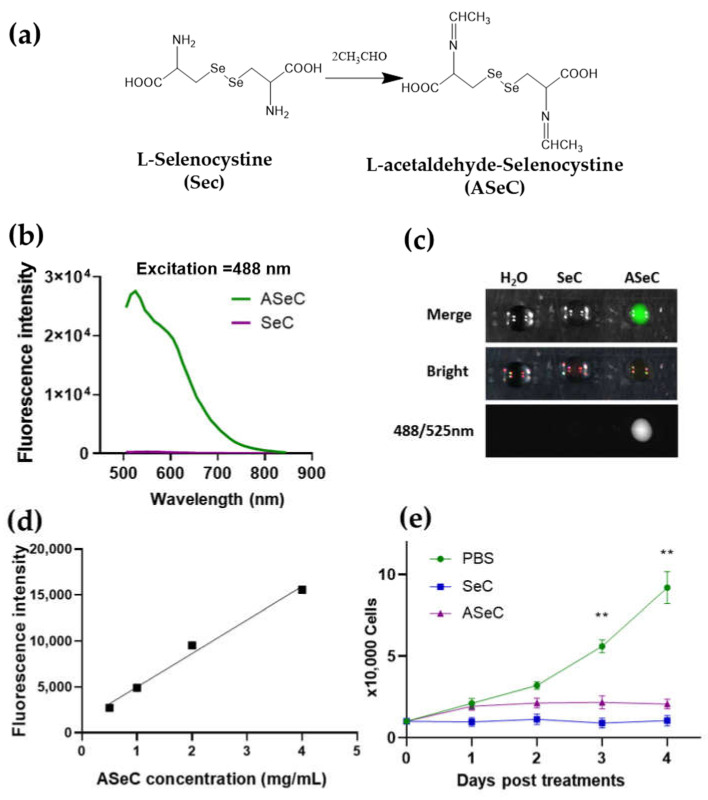
Schiff base compound derived from selenocystine reacts with acetaldehyde and exhibits fluorescent activity. (**a**) Synthesis of the acetaldehyde-selenocystine (ASeC). (**b**) ASeC fluorescence at an excitation of 488 nm. (**c**) Dot assay for detecting the fluorescence of ASeC. Fluorescence was detected in the 488/525 nm channel. (**d**) ASeC fluorescence intensity as a function of concentration. An excitation of 488 nm. (**e**) Growth curve assay for MDA-MB-231 cells treated with SeC, ASeC, or PBS control. For a single-dose treatment, 50 mM ASeC or SeC was used. *n* = 3, and data were presented as mean ± SEM. ** *p* < 0.01.

After the verification of the siRNA-loading capacity of ALC-PEI, we further moved on to explore the more novel applications of this concept. Since ALC generated by L-cystine reacted with aldehyde, the structure of L-cystine inspired us to reason that the similar structure of L-selenocystine, where the Se-Se bond replaced the S-S bond, may also exhibit fluorescence. Moreover, given that L-selenocystine was found to inhibit tumor proliferation by using the Se-Se bond in selenocystine to consume the excess protective GSH and to induce oxidative stress [9,38,39,40], the L-selenocystine-based linker may possess better treatment potential. To test this hypothesis, we first let L-selenocystine and acetaldehyde react to generate acetaldehyde-L-selenocystine (ASeC) (Figure 3a). Interestingly, we found that the ASeC indeed exhibited a fluorescent activity in the FITC fluorescent dye channel (Figure 3b,c and Figure S4), and its fluorescent intensity increased in a dose-dependent manner (Figure 3d and Figure S5). Emerging studies have reported that SeC induces reactive oxygen species (ROS) and causes cell cycle arrest and apoptosis in human cancer cells [9,38,39,40,41]. In line with these studies, the newly generated ASeC, likely due to the Se-Se bond in the compound, also imposed inhibition on the proliferation of MDA-MB-231 cells (Figure 3e).

### 2.3. siRNA Delivery Vector Development by Using Selenocystine-Derived Schiff Base

**Figure 4:**
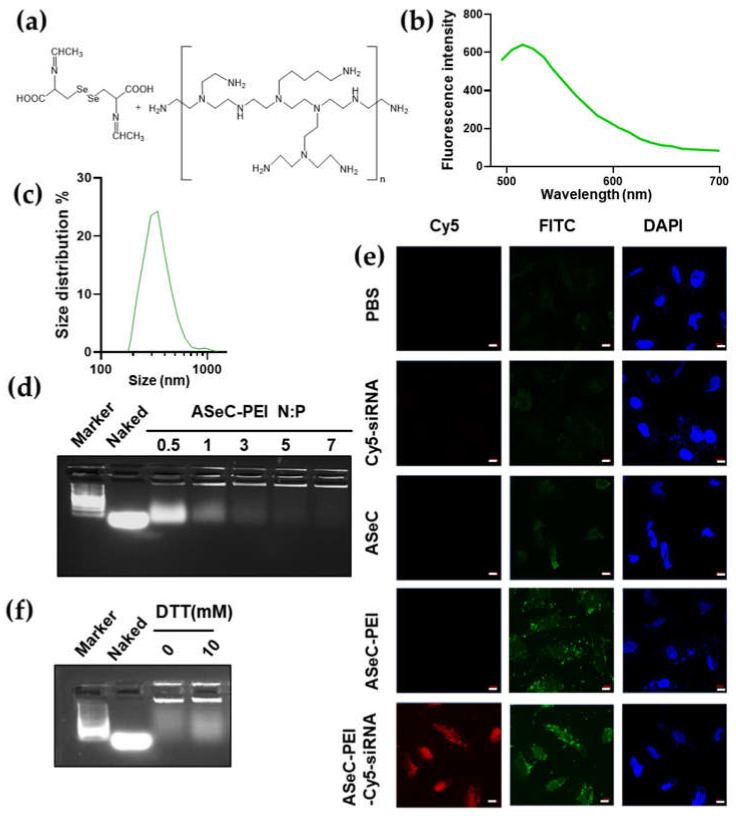
ASeC-PEI mediates siRNA loading and effective siRNA delivery. (**a**) Reaction for the structure unit in the ASeC-PEI complex. (**b**) ASeC fluorescence at an excitation of 488 nm. (**c**) Size distribution for ASeC-PEI. (**d**) ASeC-PEI siRNA loading efficiency at different N:P ratios. (**e**) Confocal imaging for Cy5-labeled siRNA (red), ASeC, and ASeC-PEI fluorescence (green). ASeC was imaged in the FITC channel. The nucleus was stained with DAPI (blue); scale bar = 10μm. Cells were treated with different conditions for 4 h and washed before imaging. (**f**) siRNA release assay under different doses of DTT treatment. N:P = 5, siRNA loaded with ASeC-PEI was incubated with DTT for 12 h before electrophoresis.

After confirming the fluorescent activities of ASeC, we next tested the application of ASeC as a linker for siRNA delivery. Similar to the ALC-PEI, we crosslinked the ASeC to the bPEI and obtained the ASeC-PEI complex (Figure 4a). The fluorescent activity of the ASeC-PEI was in line with that of ASeC (Figure 4b). Moreover, the ASeC-PEI formed a nanocomplex with a major size of around 200–500 nm (Figure 4c), and the ASeC-PEI could successfully load siRNA at an N/P ratio of five (Figure 4d). The confocal imaging analysis showed all ASeC containing groups with fluorescence in the FITC channel (green). Moreover, a co-localization for the Cy5 signal (red) and ASeC signal can be seen in the ASeC-PEI loaded with the Cy5-labeled siRNA group (Figure 4e). Data suggested an increase in the cellular uptake of the Cy5-labeled siRNA delivered by ASeC-PEI in comparison with Cy5-siRNA alone (Figure 4e). Previous studies have reported that the degradation of Se-Se could be induced by both reductive (GSH) and oxidative (H_2_O_2_) conditions [32,42]. Our data demonstrated that the ASeC-PEI achieved enhanced release both under the DTT and H_2_O_2_ stimuli (Figure 4f and Figure S6), which may enable a tumor’s microenvironment to trigger cargo release.

### 2.4. siRNA-Chemo Synergic Killing of Tumor Cells

**Figure 5:**
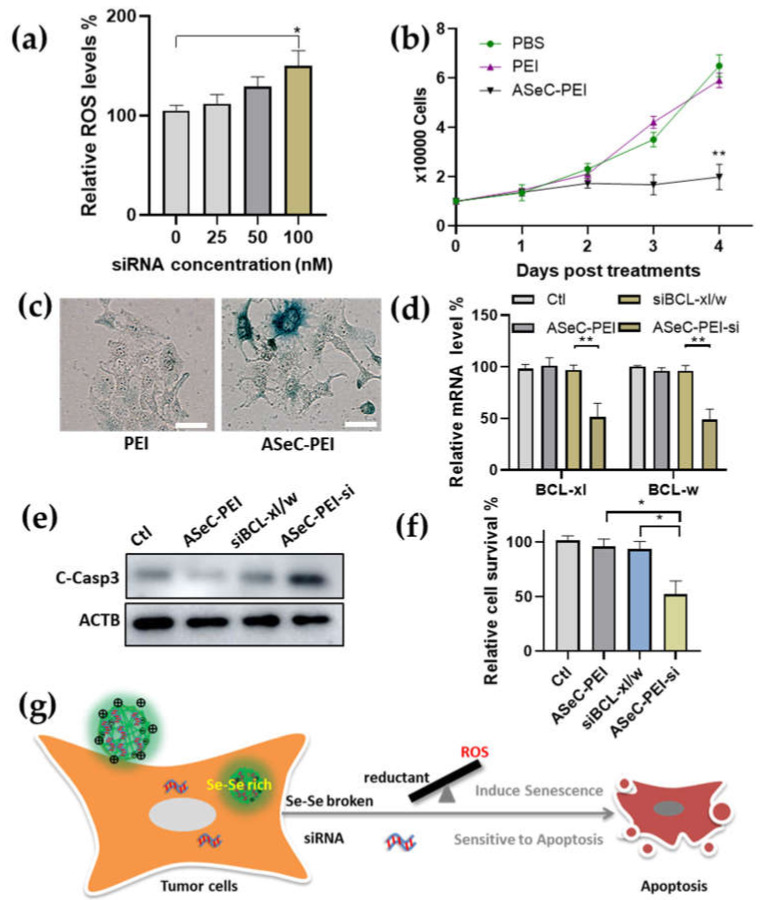
The synergic killing of tumor cells by ASeC-PEI and siRNA cocktail. (**a**) ROS probe fluorescence signal indicating ROS level in HepG2 cells was measured for scramble siRNA-loaded ASeC-PEI treatments. Dose for different treatments was referred to as scramble siRNA(nM) N:P = 5. (**b**) Growth curve assay for HepG2 cells after ASeC-PEI treatment; same conditions were used in b–f, where HepG2 cells were daily treated with ASeC-PEI, dose refers to 100 nM siRNA, N:P = 5, and samples were collected at day 5, post first treatments. (**c**) SA-b-gal staining at 5 days, post first treatments. Scale bar = 50 μm. (**d**) Relative mRNA levels after treatments. ASeC-PEI-si group was ASeC-PEI loaded with 100 nM si*BCL-xl* and si*BCL-w*. HepG2 cells were treated with different siRNA complex every day. (**e**) Cleaved caspase-3 protein expression for different groups. (**f**) Cell survival data for ASeC-PEI-siRNA groups. *n* = 3, mean ± SEM. * *p* < 0.05, ** *p* < 0.01. (**g**) A scheme to explain the synergic killing of tumor cells. siRNA was loaded onto fluorescent Schiff base linked bPEI, and the surface cation facilitated the nanocomplex entering cells. The elimination of tumor cells was initiated by a Se-Se bond broken in selenocystine-derived Schiff base linker, leading to a redox imbalance to tumor cells. Further senescence was induced to tumor cells. Meanwhile, the released siRNA targeting apoptosis-resistant genes triggered the senescent tumor cells to apoptosis.

Selenocystine has been considered to play an antitumor role due to the Se-Se bond, which depletes the intracellular GSH in tumor cells and leads to a redox imbalance [10]. Many seleno-compounds exhibit a similar function by increasing the intracellular ROS level [40]. Hence, we proposed to develop a synergic clearance of tumor cells by combining the function of the selenocystine analog and siRNA. Previous studies have revealed that an L-selenocystine treatment induces a cell cycle arrest and apoptosis of both MDA-MB-231 triple-negative breast cancer and HepG2 liver cancer cells [38,41]. Considering that ASeC-PEI is a Se-Se rich polycomplex, we first verified its proliferating inhibition capacity. Our results showed that daily treatment of scrambled siRNA, loaded with ASeC-PEI for five days, induced a higher ROS level in HepG2 cells (Figure 5a). Elevated ROS level is one of the hallmarks of cellular senescence [43]. To further verify if cellular senescence was induced, proliferating and senescence-associated β-galactosidase staining assay, a widely used senescence identification approach was performed. Cell proliferation (Figure 5b) and senescence-associated β-galactosidase staining (Figure 5c) data suggested that a senescence phenotype was induced. Considering that the direct ASeC-PEI treatment was not robust enough to kill the tumor cell in the tested concentrations but instead induced a senescence phenotype, a two-step elimination strategy was proposed. Indeed, the clearance of senescent tumor cells during chemo treatments is considered a promising new strategy for tumors [44,45]. Chemotherapy alone is usually unable to eliminate all the tumor cells and induce some tumor cells to undergo senescence. The senescent tumor cells wreck the microenvironments by secreting the senescence-associated secretory factors, which may facilitate the tumor’s progression [46,47,48,49]. Therefore, targeting chemotherapy-induced senescent tumor cells for clearance may alleviate tumor progression [47,49]. The senescent cells enter a permanent cell cycle arrest and exhibit an ability to resist apoptosis, which is largely attributed to the upregulation of anti-apoptotic *BCL-xl/w* genes. Inhibitors or siRNA targeting *BCL-xl/w* were proven to eliminate senescent cells by restoring their sensitivity to apoptosis [50,51]. Studies have also demonstrated that silencing *BCL-xl/w* can enhance chemo sensitivity [52,53,54,55,56]. Therefore, siRNA targeting *BCL-xl/w* loaded by ASeC-PEI may achieve a dual function and lead to better tumor clearance. In order to test this hypothesis, the mRNA expression for *BCL-xl* and *BCL-w* was evaluated after siRNA cocktail-loaded ASeC-PEI treatments for five consecutive days. Our results showed that si*BCL-xl/w*, loaded with ASeC-PEI, robustly silenced the gene expression and led to an increased expression of the cleaved caspase-3 concomitant with significantly induced cell death (Figure 5d–f and Figure S7).

## 3. Materials and Methods

### 3.1. Chemicals

L-cystine, L-selenocystine, acetaldehyde, and other chemicals/solvents were purchased from Macklin Lnc. (Shanghai, China). siRNA was synthesized by GenePharma. Lnc. The sequences used were from the literature: siRNA scramble control (siScr), 5′-CGUACGCGGAAUACUUCGATT[57]; *BCL-xl* siRNA sequence 5′-AUACUUUUGUGGAACUCUATT [58], *BCL-w* siRNA sequence 5′-GCAGACUUUGUAGGUUAUATT [59]. bPEI (branched Polyetherimide, molecular weight ~0.8 kDa), EDC (1-ethyl-3-(3-dimethy-laminopropyl) carbodiimide), and NHS (N-hydroxysulfosuccinimide) were purchased from Macklin Lnc. Fetal bovine serum (FBS). Trypsin-EDTA, penicillin-streptomycin (PS), DMEM cell culture medium, and phosphate buffered saline (PBS) were purchased from Gibco. TAE (tris-acetate), agarose, and Gel red were purchased from Sangon Biotechnology lnc. (Shanghai, China). 

### 3.2. Synthesis Procedures

#### 3.2.1. Synthesis of Acetaldehyde L-Cystine (ALC) and Acetaldehyde L-Selenocystine (ASeC)

L-cystine (200 mg, 0.82 mmol) was first dissolved in 50 mL of 0.1 M NaOH solution, and then, the pH was adjusted to 8 using 0.1 M HCl. Acetaldehyde (0.5 mL or 8.89 mmol) was added dropwise to the L-cystine solution over a 5 min period. The reaction mixture was stirred at room temperature overnight, then adjusted to pH 7 with 0.1 M HCl solution, and centrifuged at 10,000× *g* for 30 min thrice to remove the insoluble component. The supernatant was collected and condensed using a rotary evaporator, followed by freeze-drying to produce the ALC.

L-Selenocystine (63 mg) was dissolved in 15.75 mL 0.1 M NaOH, with an adjusted pH of 10 using 0.1 M HCl. Acetaldehyde (160 uL) was added to the L-selenocystine solution at room temperature. The reaction mixture was stirred at room temperature overnight. The supernatant was collected and condensed using a rotary evaporator, followed by freeze-drying to produce the ASeC.

#### 3.2.2. Synthesis of ALC-PEI and ASeC-PEI

In a typical synthesis of the ALC-PEI or ASeC-PEI, the NHS/EDC (0.390/0.644 mg) was added to 60 mL ALC or ASeC solution (0.005 mg/mL in deionized water) at pH 8 and stirred at room temperature for 1 h. The aqueous solution of bPEI 0.8k (1200 mg, dissolved in 12 mL deionized water) was added dropwise and then stirred in darkness at room temperature for 16 h. ALC/ASeC-PEI was dialyzed with a 3500 Da dialysis bag for 24 h and then freeze-dried. The size distribution was measured by Marlven zetasizer DLS. The unconnected ALC or ASeC to bPEI was calculated by weight loss after dialyzing and freeze-drying, and the free bPEI weight loss was deducted after the same process. This enabled calculating the control bPEI, ALC, or ASeC dose used. The Schiff base core structure was confirmed by NMR and FTIR (Figures S1 and S2).

### 3.3. Determination of Maximal Emission and Excitation for Compounds, and the Fluorescence Dot Assay

The products were dissolved in deionized water at the concentration of 1 mg/mL. The absorption spectra were measured using a UV-visible spectro-photometer (Molecular devices lnc, San Jose, CA, USA, spectraMax i3x) to determine the absorption peaks. The emission spectra were recorded with an excitation wavelength of 488 nm using a fluorescent photometer (Molecular devices lnc, spectraMax i3x). For the fluorescence dot assay, 50 μL of a different 1 mg/mL solution was added onto the transparent glass, and the photo was taken in a different channel.

### 3.4. siRNA Upload by ALC/ASeC-PEI

The polymer ALC-PEI or ASeC-PEI was dissolved in 1 mL of HEPES buffer (pH 7.4) to a stock concentration of 40 mM, and the siRNA (15 OD, 0.5 mg) was dissolved in 1 mL of HEPES buffer (pH 7.4) to a stock concentration of 1 mg/mL. The polymer solution (20 mM) was added into the siRNA solution (1 μM) (volume ratio = 1:1) and then gently pipetted and incubated for 60 min at room temperature. The N/P ratio was the amine of bPEI (N) to the phosphate of siRNA (P) ratio and represented a positive charge of bPEI and a negative charge of siRNA. To be more specific, every 330 Da (average molecular weight for nucleoside monophosphate: AMP/UMP/CMP/GMP) of siRNA contains 1 phosphate, and the average mass per positive charge for cationic nanocomplex was calculated based on the average mass per N atom in bPEI. For all cellular experiments, the siRNA and ALC/ASeC-PEI mixture N:P ratio was kept at 5 for complete siRNA encapsulation. The dose given to culturing cells was calculated using 100 nM si*BCL-w* and 100 nM si*BCL-xl* per well.

### 3.5. Gel Retardation Assay

A gel retardation assay was used to evaluate the siRNA loading capacity. The siRNA (1 mg/mL) was diluted to 1 μM using diethyl pyrocarbonate-treated water. Polymer and siRNA (1 μM) solutions (volume ratio = 1:1) were mixed at different polymer/siRNA concentration ratios; then, the mixture was gently pipetted and incubated for 60 min at room temperature. In the presence of 35 V and TAE solution, 2% agarose gel containing gel red was used to electrophoretic polymers/siRNA with different concentration ratios.

### 3.6. Cell Survival Assay

Due to the ALC, ASeC fluorescence and absorption may interrupt common CCK8 or MTT cell viability assay results, the cell survival was calculated as [1-dead cells counts/total cells counts%)], and 100,000 cells were cultured in 6-well plates and incubated with different treatments. Then, the culture medium was collected daily and replaced with a fresh medium. The collected medium containing floating trypan blue positive dead cells was centrifuged at 300 g for 3 min, and the supernatant was removed. Trypan blue positive cells in resuspension were counted as dead cells by an automatic cell counter. On the analysis day, the attached cells were trypsined, and the cell number was counted as survival cells. The total cell number was calculated by survival cell number plus dead cell number.

### 3.7. Confocal Imaging

For cellular uptake assay, cells were cultured on microscope slides in 24-well plates (1 × 10^5^ cells per sample) and incubated with different treatments in 500 μL of a medium at 37 °C for 4 h; then the culture medium was removed; and the cells were washed with PBS three times, fixed with 4% paraformaldehyde solution for 15 min, and washed with PBS three times. Then, the cell nuclei were stained with 4′,6-diamidino-2-phenylindole (DAPI) for 10 min and washed three times with PBS. The fluorescence images were obtained using a confocal microscope, Zeiss 880.

### 3.8. qPCR Assay

Total RNA was extracted using Trizol reagent. Reverse transcription and SYBR green qPCR was performed using a kit from Vazyme lnc, Nanjing China. and carried out by the Roche LightCycler 480 RT-PCR System. *β-actin* was placed as a loading control, and the delta delta Ct (2^−ΔΔCt^) method was used to calculate the fold changes. Primers were used from the literature, as shown in the following [60]: *BCL-w* F: AGT TCG AGA CCC GCT TCC R: CCC GTC CCC GTA TAG AGC *BCL-xl* F: CTG AAT CGG AGA TGG AGA CC R: TGG GAT GTC AGG TCA CTG AA *ACTB* F: GCC CTG AGG CAC TCT TCC A R: CCA GGG CAG TGA TCT CCT TCT.

### 3.9. Western Blot Assay

The cell lysate was collected by a RIPA buffer with protease inhibitor cocktail and centrifuged for 10 min (15,000 rpm, 4 °C) to collect the supernatant. After the centrifuge, the protein concentration of the supernatant was determined by using the BCA protein analysis kit. Then, the standard Western blot assay was processed. Primary antibody cleaved caspase (Abcam ab32042), β-actin (Abcam ab8226), and HRP secondary antibody (Sangon biotech. Inc. Shanghai, China.) were used.

### 3.10. Statics Assay

Results were analyzed using GraphPad Prism software, San Diego, CA, USA. Differences between two groups were assessed using unpaired *t*-tests; *n* = 3 was defined for three biological replicates. The level of statistical significance was set at *p* < 0.05. * *p* < 0.05 was considered significant, and ** *p* < 0.01 and *** *p* < 0.001 were considered highly significant. All data were expressed as mean ± SEM unless otherwise indicated.

## 4. Conclusions

Overall, this study defined the Schiff base derived from acetaldehyde, which reacted with selenocystine and exhibited a fluorescent activity with multi-application potentials. Moreover, the cleavable Se-Se bond is very useful in controlled release under redox stimuli for nanotech and biotech drug development. Future work may address the long- wavelength probe development and more accurate size control [33,61]. This study explored the potential antitumor capacity for the Schiff base analog of selenocysine. The Schiff base analog of selenocysine conjunct with PEI formed an ASeC-PEI nanocomplex that can induce the senescence of tumor cells and is capable of delivering siRNAs targeting *BCL-xl/w*. The successful *BCL-xl/w* gene silencing by the siRNA complex inhibited the apoptosis-resistant pathway in senescent HepG2 tumor cells and achieved tumor senescence syngeneic effect (Figure 5g). These findings may provide insights into developing more syngeneic strategies to target tumor cells. The simple strained Schiff base with label-free fluorescent activity may offer a broad potential for probe and traceable prodrug development.

## Data Availability

Not applicable.

## Supplementary Materials

The following supporting information can be downloaded online, Figure S1: ^1^H-NMR of ASeC and ASeC-PEI. Figure S2: Comparison on the FTIR of ASeC, ASeC-PEI. Figure S3: Cell survival for different PEI and ALC-PEI. Figure S4: Absorption for SeC and ASeC. Figure S5: Absorption of ASeC dose series at the absorption peak 488 nm. Figure S6: siRNA release assay under different doses of H_2_O_2_ treatment. Figure S7: Cell survival for ASeC-PEI loaded with scramble siRNA.

## Sample Availability

Samples of the compounds are available from the authors.
