# Supplementary material for: Selenocystine-Derived Label-Free Fluorescent Schiff Base Nanocomplex for siRNA Delivery Synergistically Kills Cancer Cells
Source: Molecules. 2022 Feb 15;27(4):1302. doi: 10.3390/molecules27041302 (PMC8878402; doi:10.3390/molecules27041302)
Supplement: Supplementary file 1 [file molecules-27-01302-s001.zip › molecules-1567396-supplementary.pdf]

Supplementary Materials

Selenocystine-Derived Label-Free Fluorescent Schiff Base Nanocomplex for siRNA Delivery Synergistically Kills Cancer Cells

Yang Liu[1], Haoying Yang[2], Qian Liu[2], Mingming Pan[2], Danli Wang[3], Shiyuan Pan[2], Weiran Zhang[2], Jinfeng Wei[2]*, Xiaowei Zhao[2*], Junfeng Ji[1]*

[1] Center of Stem Cell and Regenerative Medicine, School of Medicine, Zhejiang University, Hangzhou, 310058, China;

[2] Henan Key Laboratory of Brain Targeted Bio-nanomedicine, School of Life Sciences & School of Pharmacy, Henan University, Kaifeng, Henan, 475004, China;

[3] Zhoushan Hospital of Zhejiang Province, Zhoushan, Zhejiang, 316004, China

* Correspondence: wjf@henu.edu.cn (J.W); zxw@vip.henu.edu.cn (X.Z); jijunfeng@zju.edu.cn (J.J),

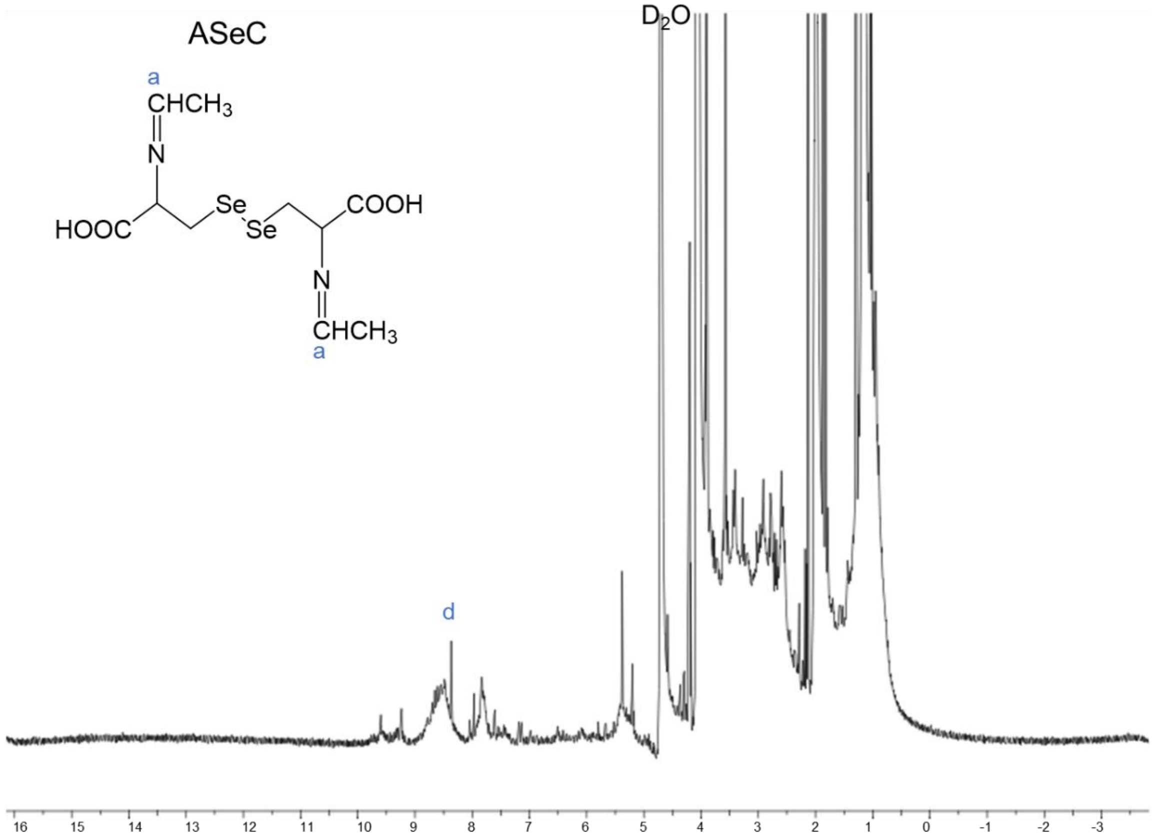
ASeC
a
CHCH3
N
HOOC
Se
Se
COOH
N
CHCH3
a
D2O
d
16 15 14 13 12 11 10 9 8 7 6 5 4 3 2 1 0 -1 -2 -3

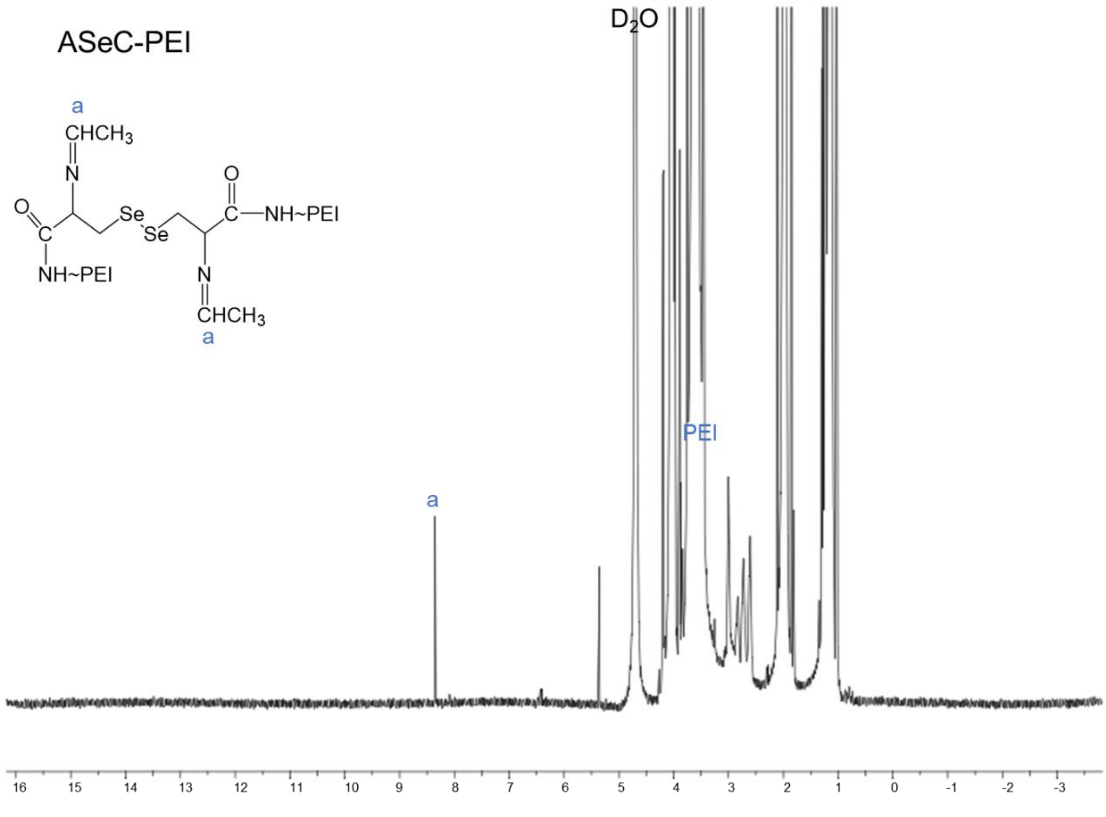
ASeC-PEI
a
CHCH3
N
O
C
NH~PEI
Se
Se
O
C
NH~PEI
N
CHCH3
a
D2O
PEI
a
16 15 14 13 12 11 10 9 8 7 6 5 4 3 2 1 0 -1 -2 -3

Figure S1. ¹HNMR of ASeC: 8.5 ppm 2H (=CHCH$_3$) in the ¹HNMR spectrum. The presence of the peak at 8.5 ppm confirms the formation of Schiff bases [36].

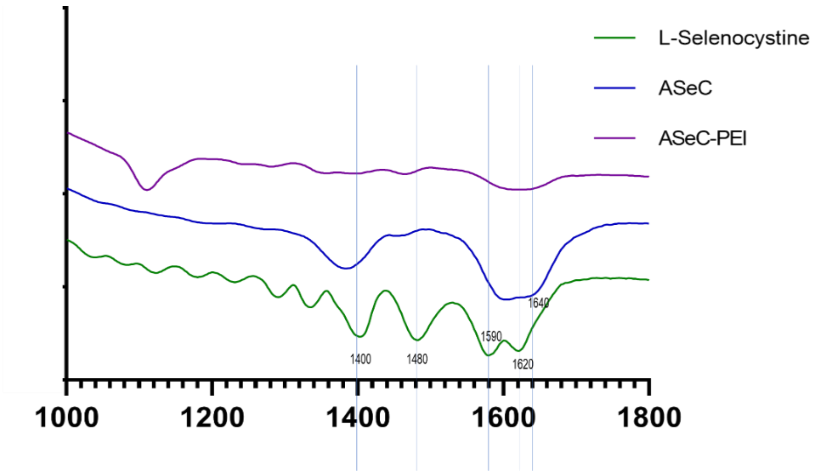

Figure S2. Comparison of the FTIR of L-selenocystine, aldehyde-L-selenocystine, and ASeC-PEI. In the FTIR spectrum of L-selenocystine, the peaks at 1590 cm^{-1} and 1620 cm^{-1} are attributed to the disulfide bond and –NH$_2$, respectively. The peak at 1640 cm^{-1} in the ASeC spectrum is also assumed to come from -N=C-. For the ASeC-PEI, the (-NH-C=O) peak at 1650 is overlaid with (-N=C-) at 1640, and thus, a broad peak is observed. The broad peak at 1630 cm^{-1} on the ASeC-PEI spectrum also contains the contribution from–NH$_2$ at 1620 cm^{-1} [36].

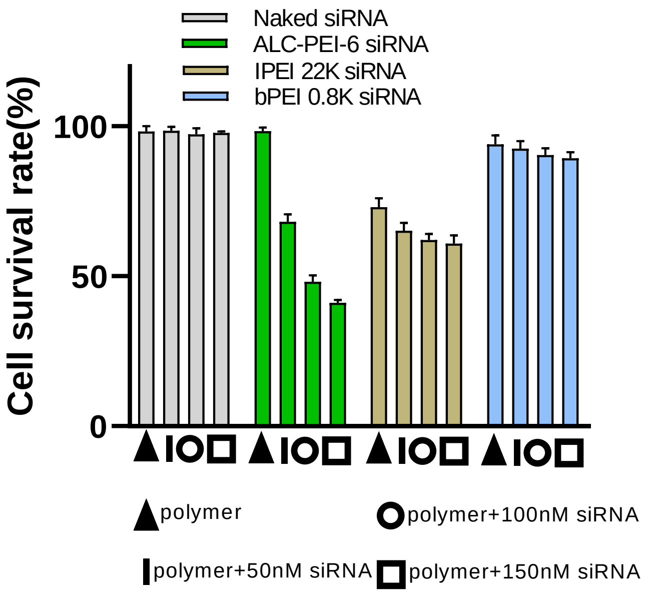

Figure S3. Cell survival rate among different treatments. PLK1 siRNA was loaded by different polymeric vectors. The data were presented as mean ± SEM. All ALC-PEI ASeC-PEI used in this study followed the ALC-PEI-6 protocol from the literature [36].

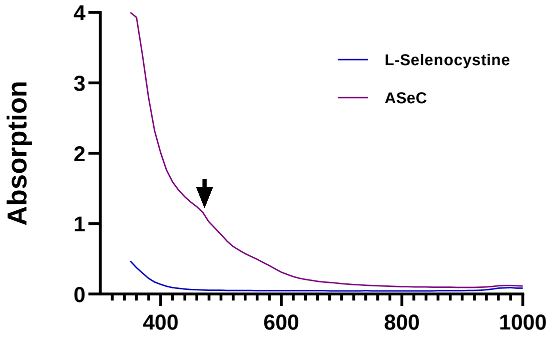

Figure S4. Absorption for SeC and ASeC.

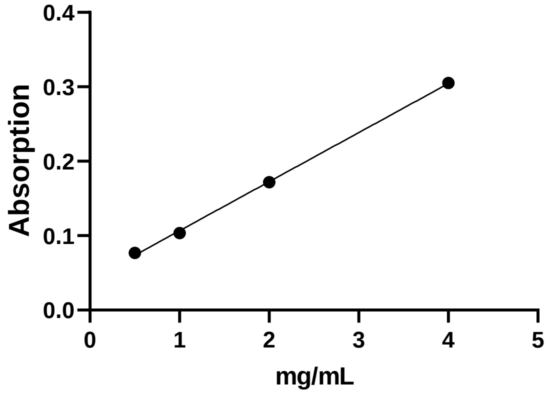

Figure S5. Absorption of ASeC dose series at the absorption peak 488 nm.

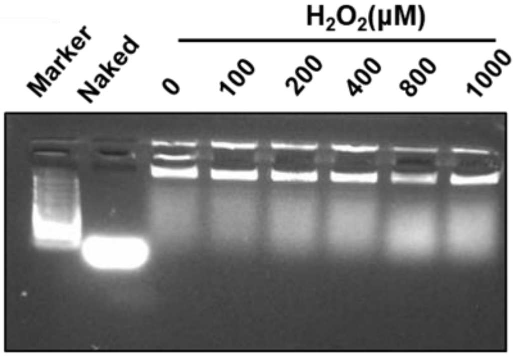

Figure S6. siRNA release assay under different doses of H_2O_2 (μM) treatment. siRNA loaded with ASeC-PEI was incubated with H_2O_2 for 12 h before electrophoresis.

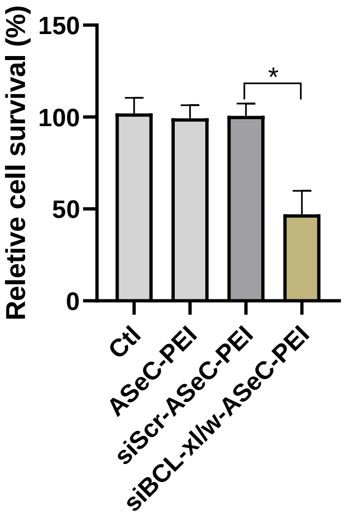

Figure S7. Cell survival for ASeC-PEI loaded with scramble siRNA. n=3, mean±SEM. *$p < 0.05$. HepG2 cells were treated with ASeC-PEI daily, dose refers to 100 nM siRNA, N:P = 5, and samples were collected at day 5 after the first set of treatments.
